# Food Fortification and Decline in the Prevalence of Neural Tube Defects: Does Public Intervention Reduce the Socioeconomic Gap in Prevalence?

**DOI:** 10.3390/ijerph10041312

**Published:** 2013-03-28

**Authors:** Mohammad M. Agha, Richard H. Glazier, Rahim Moineddin, Aideen M. Moore, Astrid Guttmann

**Affiliations:** 1 Institute for Clinical Evaluative Sciences, Toronto, ON M4N 3M5, Canada; E-Mails: rick.glazier@ices.on.ca (R.H.G.); rahim.moineddin@ices.on.ca (R.M.); astrid.guttmann@ices.on.ca (A.G.); 2 Centre for Research on Inner City Health, St. Michael’s Hospital, Toronto, M5B 1W8, Canada; 3 Pediatric Oncology Group of Ontario, Toronto, ON M5G 1V2, Canada; 4 Dalla Lana School of Public Health, University of Toronto, Toronto, ON M5T 3M7, Canada; 5 Department of Family and Community Medicine, University of Toronto, Toronto, ON M5G 1V7, Canada; 6 Department of Family and Community Medicine, St. Michael’s Hospital, Toronto, ON M5G 1V7, Canada; 7 Institute of Health Policy, Management and Evaluation, Faculty of Medicine, University of Toronto, Toronto, ON M5T 3M6, Canada; E-Mail: aideen.moore@sickkids.ca; 8 Department of Paediatrics, Faculty of Medicine, University of Toronto, Toronto, ON M5G 1V7, Canada; 9 Division of Paediatric Medicine, the Hospital for Sick Children, Toronto, ON M5G 1X8, Canada

**Keywords:** neural tube defects, food fortification, socioeconomic status, health disparities

## Abstract

*Objective*: A significant decline in the prevalence of neural tube defects (NTD) through food fortification has been reported. Questions remain, however, about the effectiveness of this intervention in reducing the gap in prevalence across socioeconomic status (SES). *Study Design*: Using health number and through record linkage, children born in Ontario hospitals between 1994 and 2009 were followed for the diagnosis of congenital anomalies. SES quintiles were assigned to each child using census information at the time of birth. Adjusted rates and multivariate models were used to compare trends among children born in different SES groups. *Results*: Children born in low SES areas had significantly higher rates of NTDs (RR = 1.25, CI: 1.14–1.37). Prevalence of NTDs among children born in low and high SES areas declined since food fortification began in 1999 although has started rising again since 2006. While the crude decline was greater in low SES areas, after adjustment for maternal age, the slope of decline and SES gap in prevalence rates remained unchanged overtime. *Conclusions*: While food fortification is successful in reducing the prevalence of NTDs, it was not associated with removing the gap between high and low SES groups.

## 1. Introduction

Despite advances in diagnosis, treatment and prevention, every year an estimated 7.9 million children worldwide are born with serious congenital abnormalities [1]. For those who survive, these disorders can cause lifelong mental, physical, auditory or visual disability. According to 2006 report [1] at least 3.3 million children less than five years of age die from congenital abnormalities each year, and an estimated 3.2 million of those who survive may have persistent disabilities. 

In Canada, nearly 11,000 newborns are diagnosed with serious congenital abnormalities every year [2]. Major congenital abnormalities remain a leading cause of death among Canadian infants in both neonatal and post neonatal periods. The prevalence rate of major birth defects overall has remained stable over the last two decades in Canada [2]. Similar findings have been reported in the U.S. [3], while in Europe [4] there has been a slight increase in overall prevalence. According to Health Canada’s Economic Burden of Illness report, the total cost (direct and indirect) associated with these anomalies in 1998 alone was an estimated $706 million [5].

In spite of a stable trend in the prevalence of major congenital abnormalities, certain defects have declined over time. There are many complexities in interpreting these changes such as, improvements in ascertainment, antenatal and newborn screening, better access to diagnostic procedures, as well as associated changes in treatment. In addition, other interventions, such as food fortification have also been proposed for the observed changes in the prevalence of some abnormalities. In Canada [2] the prevalence of anencephaly decreased from 2.2 to 0.4 per 10,000 total births between 1989 and 2002. Similarly, the prevalence of spina bifida in 2002 was 4.1 per 10,000 total births, down from 8.0 in 1989. Results of a 2009 international study [4] indicate significant declines in the prevalence of NTDs in other jurisdictions such as Atlanta (USA), England and Wales, Hungary and Japan. 

Uses of folic acid and supplements during pregnancy have been shown to be a major preventive factor for neural tube defects [6,7,8,9]. Recent reports [10,11,12,13,14,15,16] have associated food fortification with the observed decline in the prevalence of NTDs. In several countries, including the United States and Canada, recommendations to consume folic acid supplements are integrated with a public intervention of widespread fortification of flour in the US and flour, cornmeal, and pasta in Canada, to ensure that the entire population receives at least a small amount of folic acid regardless of access to supplements. 

While the birth prevalence of NTDs has declined, questions remain regarding accessibility and effectiveness of preventive measures for children born to mothers from different socioeconomic status (SES) groups. Maternal educational level and, to a lesser degree, paternal educational level and household income have been shown to be associated with the risk of giving birth to a baby with a congenital abnormality [17,18,19,20,21]. In the U.S., women with less than 10 years of schooling have a threefold increased risk of giving birth to a baby with a congenital anomaly as compared with women who have more than four years of higher education [18]. 

The SES gradient in the prevalence of NTDs could be due to mothers from low SES groups having received less benefit from preventive measures aimed at changing individual health behavior, such as taking folic acid supplements during pregnancy. For these mothers, the public intervention of food fortification may close this gap. While previous studies [9,16] have indicated the effectiveness of this intervention in reducing the prevalence of NTDs, questions remain about the effectiveness of this strategy in reducing the gap in prevalence between low and high SES groups.

The objectives of the current study were to explore the trends over time of the prevalence of NTDs among different SES groups, to investigate the relationship between the timing of food fortification and the occurrence of NTDs, and thus evaluate the effectiveness of this public health intervention in reducing the SES gap.

## 2. Methods

This cohort study was based on the follow-up of all children born in hospital in Ontario, Canada between 1994 and 2009. We accessed these data through a research agreement with the Ontario Ministry of Health and Long-Term Care. Approval for this study was granted by the Research Ethics Board of Sunnybrook Health Sciences Centre in Toronto, Canada.

The following databases were used to assemble the cohorts and collect data. Information on hospital admissions was obtained from the Discharge Abstract Database of the Canadian Institute for Health Information (CIHI-DAD), regardless of where in Ontario the hospitalization took place. This database includes newborn date of birth, infant sex, and postal code at the time of birth, encrypted unique health care number, admission dates and the diagnoses and procedures coded at hospital discharge. The reliability and validity of this database have been documented [22,23]. The Registered Persons Database (RPDB) is Ontario’s population-based health care registry, which provides basic demographic information (date of birth, date of death, address changes) about those who ever received a health care number in Ontario. 

All these databases are linkable through a unique identifier (health care number) assigned to each individual by Ontario Ministry of Health and Long-Term Care. Health care numbers are scrambled in the research version of these databases which stripped off identifying information.

All children born alive in hospital in Ontario during 1994–2009 inclusive were identified. Using the child’s encrypted health care number as a unique identifier and through record linkage, their hospital birth records were linked to RPDB and CIHI-DAD in order to collect records of their hospitalizations during the first year of life. 

Children who met the following criteria were considered to have congenital abnormalities: (1) born alive between 1994 and 2009 in a hospital in Ontario to mother’s residing in Ontario and; (2) had at least one diagnosis in the CIHI-DAD from the Congenital Anomalies chapter of the International Classification of Diseases (ICD) during their first year of life. All ICD9 codes between 7,400 and 7,420 and their equivalent in ICD10 (from 2002 on) were considered NTDs. There was no major change in the classification of cases or in the way children were hospitalized during this timeframe.

Canadian census data on income, education level, and other indicators at the smallest geographic level available were used to assign area level socioeconomic indicators to each child. Socioeconomic status levels are area-based indicators generated from census data for enumeration or dissemination areas. Each enumeration area (EA) includes socio-demographic information for an average of 282 households. During the study period, Statistics Canada changed the definition of EA to dissemination area (DA) while maintaining similar size and homogeneity. In order to assign children to one of these areas we used the closest census to their birth year, *i.e.*, EA using the 1996 census or DA using the 2001 and 2006 Canadian censuses. The most recent postal code conversion file, PCCF+ 2006, was used in EA or DA assignment. 

We used the following processes for SES assignment, (1) Using hospital discharge data (CIHI) all children born in Ontario and their encrypted health care number were identified. The encrypted health care number was used to link each child to RPDB to identify their postal code at the time of birth and date of death (if applicable), (2) postal code was used to link each child to an EA or DA based on census definition, and (3) census information for each area was used to assign a SES quintile to each child.

Using the household information in each EA or DA, income and education quintiles were generated and assigned to each child with 1 being the lowest and 5 being the highest. For income, neighborhood income per person equivalent (IPPE) was used. This is a community and household size-adjusted measure of household income. 

The measure used for congenital abnormalities was birth prevalence, representing the number of malformations detected at birth or during the first year of life per 1,000 live births per calendar year.

For prevalence within each quintile of SES, the number of anomalies for each quintile was divided by number of births within quintiles. 

Annual prevalence rates were smoothed using the moving average method [24]. Prevalence for each year was added to the prevalence in previous and following years and then averaged. The averaging process was based on 50% weight for the current year and 25% weight each for the previous year and the following year. Also, in order to improve clarity in reporting data in some graphs only, and to avoid small sample size issues for specific anomalies, quintiles 1 and 2 were grouped as low income areas and quintiles 4 and 5 as high income areas.

We used direct standardization to generate standardized (for maternal age in ten-year age groups) rates for prevalence of NTD in low and high income groups during 1994–2009. The population of all children born in Ontario during this period (1994-2009) was used as the standard population. 

We used multivariate logistic regression to generate the odds ratios for being born with NTD while adjusting, a priori, for a limited number of covariates including infant sex, maternal age, neighborhood income, and neighborhood education. 

Prenatal screening and pregnancy termination are major contributing factors to the birth prevalence of NTDs. Unfortunately, lack of access to this data in Ontario is one of the limitations of this study and it meant we could not control for these factors. As a surrogate measure, we used Trisomy 21 as a comparison defect to examine the possible role of these factors. Like NTD’s, trisomy 21 may be diagnosed early in pregnancy through screening. We considered the differences in the maternal age-adjusted birth prevalence of this condition between low and high SES groups over time to be attributable to differential use of screening and pregnancy termination. 

Food fortification started in Ontario in 1999 [9]. To test whether rates of NTD’s prior to food fortification (1994–1999) were different from those after fortification; a binary indicator for before and after that year was included in regression models. In order to test if the effect of income on developing NTD was different before and after food fortification, an interaction between food fortification year indicator and income was examined in the regression models. All analyses and data manipulations were done using SAS version 9.2.

## 3. Results

A total of 2,152,750 children were born in Ontario between 1994 and 2009. More than 23% of these children were born in families residing in low income areas (Q1), and 16% in high income (Q5) areas (*P* < 0.001). The mean maternal age for these children was 29.5 years. Children born in high income areas (Q5) had a significantly higher mean maternal age than those born in low income (Q1) areas (31.1 years *vs*. 27.8, *P* < 0.001). 

Among these children, 144,321 were diagnosed with congenital anomalies during their first year of life. Table 1 shows the yearly prevalence for NTD and some characteristics for all children born with any congenital anomalies in Ontario. During the study period the proportion of mothers over 35 years of age at the delivery of children with anomalies increased by nearly 70% from 13.4% in 1994 to 22.7% in 2009. 

**Table 1:** Birth prevalence (/1,000) of Neural Tube Defects (NTD) in Ontario, 1994-2009.

		All Children with Congenital Abnormalities				
Birth Year	Number of Births	% Male	%With Mother ≥Wi Years	% VLBW *	# of Infants with NTD	Prevalence of NTD
**1994**	144,259	57.6	13.4	6.5	365	2.53
**1995**	140,413	57.2	14	6.9	333	2.37
**1996**	134,186	57.6	16.3	7	345	2.57
**1997**	131,676	56.6	15.9	6.1	307	2.33
**1998**	130,066	56.7	16.8	6.1	316	2.43
**1999**	128,024	56.8	18.2	6.5	328	2.56
**2000**	126,260	57.1	19.1	6.4	304	2.41
**2001**	131,723	57.7	18.9	6.5	325	2.47
**2002**	128,812	57.7	19.5	6.3	304	2.36
**2003**	131,481	59.2	20.6	7.5	326	2.48
**2004**	133,604	60.1	20.7	7.6	287	2.15
**2005**	134,611	60.3	21	8.7	295	2.19
**2006**	136,751	60.4	20.8	8.5	264	1.93
**2007**	141,085	60.2	21.9	8.7	340	2.41
**2008**	139,855	61.3	23	8.6	324	2.32
**2009**	139,944	60.6	22.7	9.1	371	2.65
**Total**	2,152,750	58.5	18.9	7.2	5,145	2.39

* VLBW: very low birth weight.

Nearly 7% children born with anomalies had very low birth weight (<1,500 grams). This rate increased from 6.5% in 1994 to 9.1% by 2009.

Table 2 shows the prevalence of NTDs by SES indicators. Children born in low income areas had significantly higher rates of NTD (RR = 1.29, CI: 1.15–1.34) as compared to those born in high income areas.

**Table 2:** Birth prevalence (/1,000) of Neural Tube Defects (NTD) by socioeconomic indicators, Ontario 1994-2009.

Quintiles		Mean Maternal Age	N	Prevalence NTD
Household Income				
	Q1 = Lowest	27.8	1,330	2.72
	Q2	28.7	1,047	2.43
	Q3	29.4	949	2.23
	Q4	30.4	882	2.08
	Q5 = Highest	30.9	733	2.10
% With University Degree				
	Q1 = Lowest	27.4	1,072	2.61
	Q2	28.6	1,007	2.45
	Q3	29.3	973	2.25
	Q4	30.2	939	2.22
	Q5 = Highest	31.5	827	2.09

The prevalence of NTD was also associated with educational attainment (Table 2). Children born in areas with fewer people having university degrees had a significantly higher prevalence of NTDs (RR = 1.25, CI: 1.14–1.37).

**Figure 1:**
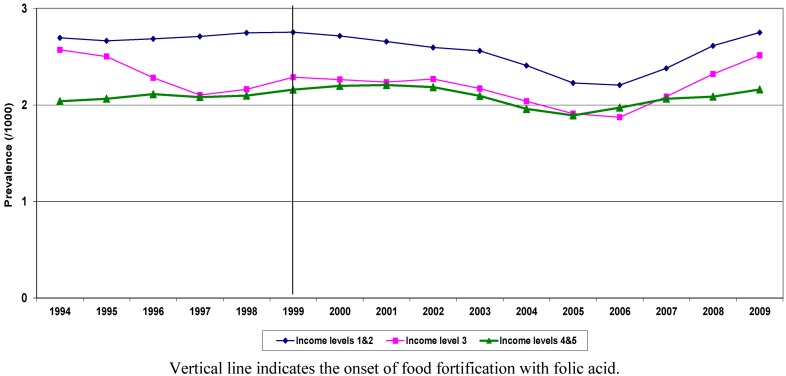
Crude birth prevalence of NTD by income quintiles, Ontario, 1994-2009.

Figure 1 shows the trend in birth prevalence of NTDs by income quintiles. NTDs showed a declining trend from 1999. The rate of reduction was larger for children born in lower income quintiles (Q1, Q2). 

**Figure 2:**
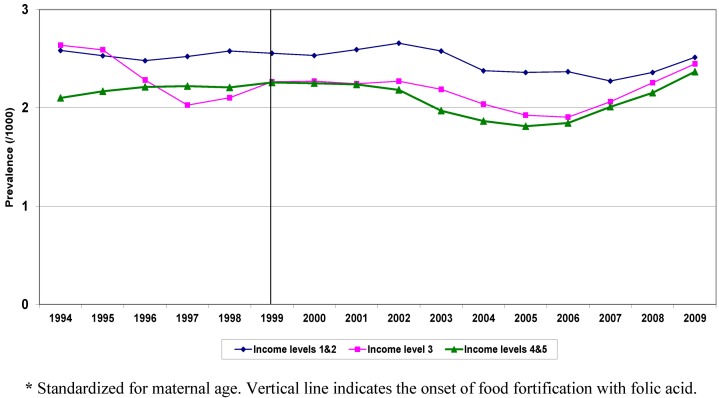
Standardized * Birth Prevalence of NTD by Income Quintiles, Ontario, 1994-2009.

NTD prevalence in Q1, Q2 declined from 2.7/1,000 in 1999 to 2.1/1,000 in 2006 (21% reduction), while in Q4, Q5, during the same period, the decline was 11%. In both groups, the rates started rising again in 2007. Children born with NTDs in low and high income groups also differed with respect to other risk factors, such as maternal age and area level education. The prevalence of these factors changed during the study period for both groups. Figure 2 shows the trend over time in standardized (for maternal age and education) prevalence for NTD by income quintiles. The advent of food fortification is flagged in the graph, showing 1999 as the start year for this public intervention.

Standardized rates began to decline in all SES groups in the early 2000s. Standardization for maternal age and education indicates that the slope of the decline among low and high income groups remained unchanged over time. 

**Figure 3:**
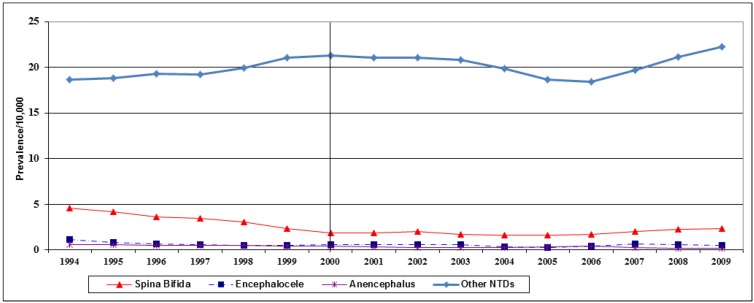
Birth prevalence (/10,000) of NTD sub-categories in Ontario, 1994-2009.

Compared to Q4, Q5, children born in Q1, Q2 had higher rates during the study period. Both before and after food fortification, children born in low income areas were more likely to be born with a NTD.

Figure 3 shows the trend over time for the major subgroups of NTDs. While eniencephaly and Encephalocele did not show any substantial decline over time, the decline in spina bifida began at the start of study period in 1994. 

**Figure 4:**
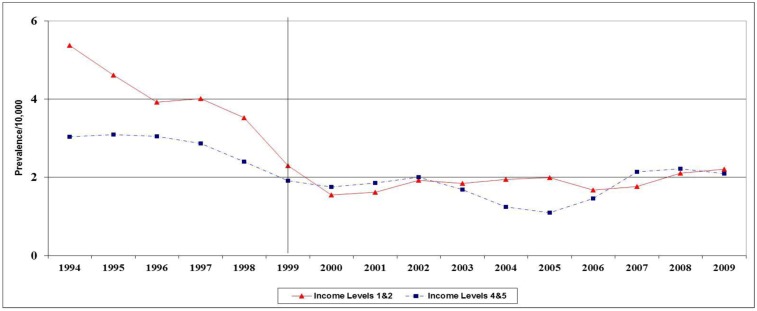
Birth prevalence (/10,000) of spina bifida by income quintiles in Ontario, 1994-2009.

Comparing birth prevalence of spina bifida in low (Q1, Q2) and high (Q4, Q5) income groups indicates that the decline in both groups started before 1999 (Figure 4), while the prevalence in low and high SES groups were similar after 1999. 

Table 3 shows the results of regression analysis. Low education and low income level remained statistically significant risk factors for NTD after adjustment for maternal age, infant sex and time era. Rates were significantly lower in the late era. There was no statistically significant interaction between income and time (*p* = 0.85).

**Table 3:** Adjusted odds ratios for maternal and infant characteristics, Ontario 1994-2009.

Variables	Levels	Adjusted OR	95% CI
Sex	Female *vs*. Male	0.88	0.83–0.94
Time era	Pre 1999 *vs*. After 1999	1.06	1.00–1.24
Maternal age	<20 *vs*. 20–29 years	1.33	1.16–1.51
	30–39 *vs*. 20–29 years	1.01	0.95–1.07
	≥40 *vs*. 20–29 years	1.29	1.05–1.59
Income quintile	1 *vs*. 5	1.20	1.08–1.33
	2 *vs*. 5	1.08	0.97–1.20
	3 *vs*. 5	1.06	0.91–1.12
	4 *vs*. 5	0.96	0.87–1.07
University degree	1 *vs*. 5	1.17	1.05–1.30
	2 *vs*. 5	1.14	1.03–1.26
	3 *vs*. 5	1.05	0.95–1.16
	4 *vs*. 5	1.07	0.97–1.18

## 4. Discussion and Conclusions

In this population-based study, we observed declining trends in the prevalence of NTDs since 1999 but with rates rising again after 2006. Among NTDs, prevalence for anencephalus and iniencephaly remained stable over time while spina bifida declined considerably, even prior to food fortification. The current study indicates a consistent SES disparity in the birth prevalence of NTDs in Ontario across time. During 1994-2009, the prevalence of all NTDs combined, as well as specific ones, remained higher in low SES areas. Children born in low SES areas had a more than 29% higher risk of having a NTD. 

Our results indicate a 21% decline (from 1999 to 2006) in the prevalence of NTDs in Ontario associated with the timing of food fortification. For specific NTDs, such as spina bifida, the decline started earlier than the intervention, likely as a result of folic acid and multivitamin supplements use. 

Our results are comparable with others [9,13,14,15] in describing an overall decline in NTD’s over time, especially after food fortification. Although we show that all the declines were sustained by women across all SES strata there was no significant narrowing of the gap between high and low income areas. Although the decline in the crude prevalence of NTD was higher among low SES groups (21% in low income quintiles *vs*. 11% in high income quintiles), after adjustment for maternal age and education the slope of the decline was the same. There was no statistically significant interaction between income and the intervention time flag in the regression model. This means that although food fortification as a public intervention for reducing the prevalence of NTDs was successful, it was not effective in eliminating the disparity in NTDs by SES groups, at least in the first 6 years after food fortification.

The pioneering studies of Smithells *et al*. [25] and others [6,7,8] showed preconception and prenatal use of folic acid-containing multivitamin supplementation reduces NTDs. This resulted in pre-pregnancy counseling initiatives and, later, food fortification interventions in many countries, including Canada in 1999. Interventions tailored for the general population (such as immunization, food fortification and screening) have been considered one of the main solutions for reducing disparities between low and high SES populations. Food fortification is considered one of the success stories in the fight against congenital abnormalities and specifically NTDs. The nature of this intervention, that is, mandatory supplementation of flour and other wheat products with folic acid provides universal and free access for all pregnant women from all social and economic classes. The Canadian milling industry started fortification early in 1997 [26]. The mandatory level of folic acid in flour was set to be 0.15 mg per 100 g of flour. This is very similar to the amount used by the US in order to match practice between two countries [26]. 

Although one might expect food fortification to result in a greater reduction of NTDs in low SES areas (due to lower level of access and knowledge about folic acid consumption before public intervention), we did not find a steeper decline in these areas after food fortification. This is the first study to suggest that such public intervention is not enough to eliminate the gap among SES groups. Reducing disparities in preventable congenital abnormalities may require additional strategies.

There is no doubt that health disparity is a major burden for any society and a health system cost driver. According to a recent report [27] approximately 20% of total health care spending in Canada may be attributable to SES disparities. Few studies have investigated the association between SES and congenital abnormalities [17,18,19,20,21] and these indicate that maternal and paternal educational levels and household income are associated with the risk of giving birth to a baby with a congenital anomaly.

While a mechanism for the association is still unknown, some postulated that physical aspects of the living conditions, such as environmental factors, quality of housing, violence, availability of food, relate to the risk of having a child with NTD [18].

The minor increase in prevalence of spina bifida we show in Ontario from 2006 is also seen in some recent international surveillance reports [28]. Reasons for this remain unclear and require further investigation, but may be related to increasing maternal obesity and its acknowledged link with neural tube defects [29].

Other possible mechanisms could be responsible for the observed SES gradient in the birth prevalence of NTD, including differential uptake of prenatal care and screening by different SES groups. More detailed prenatal screening may lead to higher rates of pregnancy termination. Unfortunately, limitations in our data, including lack of access to pregnancy termination data and prenatal screening do not permit us to control for the possible effect of these factors. However, our finding on the trend in Trisomy 21 suggests that prenatal screening may not be an explanatory factor. More research is needed to study the possible role of other factors such as pregnancy termination. 

Another limitation is lack of access to data such as vitamin and other supplement intake by mothers during pregnancy based on their SES status. Higher intakes of supplements by more educated and wealthier mothers could be one of reasons for their lower risk. 

This study relied on hospital records ICD codes to identify birth defects cases. In doing so, issues such as coding, transcription and misclassification errors are possible. We only had access to anonymous database and we had no means to confirm the birth defect status of the child independently.

Future studies need to explore the contribution of other factors responsible for the higher prevalence of congenital abnormalities among low SES groups, including dietary and other behaviors during pregnancy, access to health care, diagnostic services and pregnancy termination. 

Identification of these factors and their incorporation into public health and clinical policies and practices should lead to further decreases in the prevalence of congenital anomalies and reduction in disease disparity.

## References

[1] Christianson A., Howson C.P., Modell B. (2006). The March of Dimes Global Report on Birth Defects: The Hidden Toll of Dying and Disabled Children.

[2] Health Canada (2002). Congenital Anomalies in Canada: A Perinatal Health Report, 2002.

[3] Centers for Disease Control and Prevention (2008). Update on overall prevalence of major birth defects—Atlanta, Georgia, 1978–2005. Morb. Mortal. Wkly Rep..

[4] Dolk H., Loane M. The Status of Health in the European Union: Congenital Malformations 2009.

[5] Health Canada (2002). Economic Burden of Illness in Canada, 1998.

[6] MRC Vitamin Study Research Group (1991). Prevention of neural tube defects: Results of the medical research council vitamin study. Lancet.

[7] Czeizel A.E., Dudas I. (1992). Prevention of the first occurrence of neural-tube defects by periconceptional vitamin supplementation. N. Engl. J. Med..

[8] Berry R.J., Li Z., Erickson J.D., Song Li., Moore C.A., Wang H., Mulinare J., Zhao P., Wong L.Y., Gindler J. (1999). Prevention of neural-tube defects with folic acid in China. N. Engl. J. Med..

[9] De Wals P., Tairou F., van Allen M.I., Uh S., Lowry R.B., Sibbald B., Evans J.A., van den Hof M.C., Zimmer P., Crowley M. (2007). Reduction in neural-tube defects after folic acid fortification in Canada. N. Engl. J. Med..

[10] Rosano A., Smithells D., Cacciani B., Castilla E., Cornel M., Erickson D., Goujard J., Irgens L., Merlob P. (1999). Time trends in neural tube defects prevalence in relation to preventive strategies: An international study. J. Epidemiol. Community Health.

[11] Besser L.M., Williams L.J., Cragan J.D. (2007). Interpreting changes in the epidemiology of anencephaly and spina bifida following folic acid fortification of the U.S. grain supply in the setting of long-term trends, Atlanta, Georgia, 1968–2003. Birth Defects Res. A Clin. Mol. Teratol..

[12] De Wals P., Tairou F., van Allen M.I., Lowry R.B., Evans J.A., van den Hof M.C., Crowley M., Uh S.H., Zimmer P., Sibbald B. (2008). Spina bifida before and after folic acid fortification in Canada. Birth Defects Res. A Clin. Mol. Teratol..

[13] Williams L.J., Rasmussen S.A., Flores A., Kirby R.S., Edmonds L.D. (2005). Decline in the prevalence of spina bifida and anencephaly by race/ethnicity: 1995-2002. Pediatrics.

[14] Honein M.A., Paulozzi L.J., Mathews T.J., Erickson J.D., Wong L.Y. (2001). Impact of folic acid fortification of the US food supply on the occurrence of neural tube defects. JAMA.

[15] Canfield M.A., Collins J.S., Botto L.D., Williams L.J., Mai C.T., Kirby R.S., Pearson K., Devine O., Mulinare J. (2005). Changes in the birth prevalence of selected birth defects after grain fortification with folic acid in the United States: Findings from a multi-state population-based study. Birth Defects Res. A Clin. Mol. Teratol..

[16] Williams L.J., Mai C.T., Edmonds L.D., Shaw G.M., Kirby R.S., Hobbs C.A., Sever L.E., Miller L.A., Meaney F.J., Levitt M. (2002). Prevalence of spina bifida and anencephaly during the transition to mandatory folic acid fortification in the United States. Teratology.

[17] Olesen C., Thrane N., Ronholt A., Olsen J., Henriksen T.B. (2009). Association between social position and congenital anomalies: Population-based study among 19,874 Danish women. Scand. J. Public Health.

[18] Wasserman C.R., Shaw G.M., Selvin S., Gould J.B., Syme S.L. (1998). Socioeconomic status, neighborhood social conditions, and neural tube defects. Am. J. Public Health.

[19] Farley T.F., Hambidge S.J., Daley M.F. (2002). Association of low maternal education with neural tube defects in Colorado 1989–1998. Public Health.

[20] Rosano A., Del Bufalo E., Burgio A. (2008). Socioeconomic status and risk of congenital malformations. Epidemiol. Prev..

[21] Yang J., Carmichael S.L., Canfield M., Song J., Shaw G.M. (2008). Socioeconomic status in relation to selected birth defects in a large multicentered US case-control study. Am. J. Epidemiol..

[22] Goel V., Williams J., Anderson G., Blackstien-Hirsch P., Fooks C., Naylor D. (1996). Appendix: A summary of studies on the quality of health care administrative databases in Canada. Patterns of Health Care in Ontario. The ICES Practice Atlas.

[23] Juurlink D., Preyra C., Croxford R., Chong A., Austin P., Tu J., Laupacis A. (2006). Canadian Institute for Health Information Discharge Abstract Database: A Validation Study.

[24] NIST/SEMATECH e-Handbook of Statistical Methods. http://www.itl.nist.gov/div898/handbook.

[25] Smithells R.W., Sheppard S., Schorah C.J., Seller M.J., Nevin N.C., Harris R. (1980). Possible prevention of neural-tube defects by preconceptional vitamin supplementation. Lancet.

[26] Flour Enrichment. http://www.canadianmillers.ca/flour_enrichment.htm.

[27] Canadian Institute for Health Information (CIHI) (2008). Reducing Gaps in Health: A Focus on socioeconomic status in urban Canada.

[28] (2010). International Clearinghouse for Birth Defects Surveillance and Research—Annual Report;.

[29] Rasmussen S.A., Chu S.Y., Kim S.Y., Schmid C.H., Lau J. (2008). Maternal obesity and risk of neural tube defects: A metaanalysis. Am. J. Obstet. Gynecol..

